# Concerning Increase in Antimicrobial Resistance Patterns of Pathogenic Strains of *Salmonella* Isolated in Poultry Meat Products

**DOI:** 10.3390/antibiotics11111469

**Published:** 2022-10-25

**Authors:** Anca Forgaciu, Alexandra Tabaran, Liora Colobatiu, Romolica Mihaiu, Sorin Daniel Dan, Marian Mihaiu

**Affiliations:** 1 Department of Animal Breeding and Food Science, Faculty of Veterinary Medicine, University of Agricultural Sciences and Veterinary Medicine, Manastur Street No. 3/5, 400372 Cluj-Napoca, Romania; 2 Department of Medical Devices, Faculty of Pharmacy, Iuliu Hatieganu University of Medicine and Pharmacy, Victor Babes Street No. 8, 400012 Cluj-Napoca, Romania; 3 Department of Management, Faculty of Economic Sciences and Business Administration, Babes Bolyai University, Mihail Kogalniceanu Street No. 1, 400084 Cluj-Napoca, Romania

**Keywords:** antimicrobial resistance, resistance genes, *Salmonella*, poultry meat

## Abstract

*Salmonella* is considered to be one of the major foodborne pathogens associated with the consumption of contaminated poultry meat products. To the best of our knowledge this is the first extended research performed on a number of *Salmonella* strains isolated during 2011–2021 from poultry meat products in Romania. The aim of this study was to characterize the prevalence of pathogenic *Salmonella* serovars, antimicrobial susceptibility, and antimicrobial resistance genes in 112 *Salmonella* isolates recovered from raw poultry meat products. The results showed that *Salmonella enterica* serovars Enteritidis and Typhimurium were the common serotypes (56%; 25%). Overall, the majority of the isolates were resistant to at least three tested antimicrobials. High resistance was observed for tetracycline (84%), nalidixic acid (78%), and ampicillin (78%) in pathogenic *Salmonella* isolated during the period 2016–2021. All the pathogenic *Salmonella* isolated during 2016–2021 tested positive to at least one resistance gene encoding for tetracycline resistance, with the *tetA* gene being the most prevalent (62%). In addition, 64% (24/37) of the *Salmonella* isolates carried at least one of the genes (*blaCMY-2, blaSHV1, blaTEM1*) that code for β-Lactams resistance. The findings in this study showed a high prevalence of multi-drug resistant (MDR) *Salmonella* serovars in poultry meat products and a concerning increase of resistance patterns. The continuous occurrence of more resistant strains implies that effective measures should be strictly applied in this particular food chain in order to prevent their spread and guarantee microbial safety.

## 1. Introduction

*Salomonella* infection is considered to be among the most widespread foodborne bacterial illness in humans [1,2]. Its clinical manifestations in an infected population can vary from mild to severe. The most common clinical presentation of *Salmonella* infection is gastroenteritis, which can be caused by more than 150 *Salmonella* serotypes, known as non-typhoidal *Salmonella* (NTS) [3].

The main cause of contamination with pathogenic *Salmonella* is considered to be contaminated foods such as undercooked meat, eggs, dairy products, as well as fresh vegetables [2,4]. Particularly, poultry meat has been incriminated to be among the most susceptible meat product in the case of *Salmonella* presence, and that is one of the main reasons for which a surveillance system is mandatory in all bird slaughtering units [5]. The Center for Disease Control and Prevention (CDC) estimates that *Salmonella* causes more foodborne illnesses than any other bacteria [6]. It is a growing incidence due to the increased industrialization of the food supply and globalization of processed and ready to eat food [1,2,7]. In Europe, the growing incidence of *Salmonella* was seen starting with 2013 when isolates were found in egg laying hens [8]. In 2021, France has reported an increase in *Salmonella* *enteritidis* infections and by 2022 the number of cases has spread to other European countries [9]. There were 272 confirmed cases that resulted in 2 deaths and 25 patients hospitalized [9]. In Romania particularly, the incidence of *Salmonella* has shown a growing trend, the latest outbreak being caused in 2021 by the consumption of contaminated poultry organs (hearts and liver).

Unfortunately, nowadays, it is not only the presence of *Salmonella* that holds concerns but also its multi-drug resistance profile. This growing trend of resistant bacteria is most likely due to the frequent use of antibiotics in animal growing systems [10]. Another role in an acquired resistance il played by plasmids which frequently act as vehicles for horizontal gene transfer [11]. More so, the coadaptation between bacterial hosts and plasmids frequently results in adaptive changes restricted exclusively to host genome leaving plasmids unchanged [12], and this is why studies that underline the possible effects of such genetic transfers are crucial in limiting the spread. During 2009–2011, a worrying percentage (5) of nontyphoidal *Salmonella* were resistant to ≥5 types of drugs [13]. In 2015, among 2364 *Salmonella* isolates tested, 65 of them (2.7%) were resistant to at least 5 antimicrobial agents: ampicillin, chloramphenicol, streptomycin, sulfonamide and tetracycline. *Salmonella typhimurium* was the serotype that most frequently presented ampicillin, chloramphenicol, streptomycin, sulfonamide and tetracycline resistance with a prevalence of 10.8% (27 of 251) [14].

A number of studies have shown that during the last 20 years, *Salmonella* strains isolated from various food products have become highly resistant to several classes of antibiotics, which were normally used as first intention treatment in animal bacterial diseases [15]. This fact has become a worldwide public health concern, since the therapeutic options available are becoming scarcer [16]. That is why the proper and accurate surveillance of antimicrobial resistance profiles is essential in providing the data necessary for a correct evaluation of the resistance magnitude levels and efficient treatment protocols.

## 2. Results

### 2.1. Prevalence of Pathogens in Salmonella Isolates

The polymerase chain reaction (PCR) for the identification of *S. enteritidis* and *S. Thyphimurium* was performed on all *Salmonella* isolates (*n* = 112) found between 2011–2021. We found that the majority of the samples, 56% were positive for *S. enteritidis* (*n* = 63), 25% belonged to *S. Thyphimurium* (*n* = 28) and the rest of the isolates were negative for both strains (*n* = 21). Regarding the prevalence during the period studied (2011–2021), we found that during the years 2011–2015 the largest number of pathogens were found (*n* = 54; *S. enteritidis*—38; *S. Thyphimurium*—16). In the period 2016–2021, 37 samples were identified as *S. enteritidis* (*n* = 24) and *S. Thyphimurium* (*n* = 13).

### 2.2. Antimicrobial Susceptibility

The results of the antimicrobial resistance test for the 91 pathogenic *Salmonella* isolates during a ten-year period of study have shown a concerning increasing in resistance. The isolates that tested resistant to three or more antimicrobials were classified as multidrug resistant (MDR). Out of the 91 *Salmonella* isolates, a large number (*n* = 78) were found to be MDR. Concerning was also the fact that during the period studied, all the isolates found in poultry meat products were resistant to at least two antimicrobials. None of the isolates studied showed sensitivity to all the antimicrobials tested.

The results on the antimicrobial susceptibility of the pathogenic *Salmonella* isolates during the period 2011–2015 have shown that the most prevalent resistance phenotype was to older antimicrobials such as TET (74%), sulfonamide (54%), S (34%), and AMP (32%) (Table 1). Most of the isolates remained susceptible to cephalosporins and quinolones with 8% and 4%, respectively, showing a resistance phenotype. We also found that *S. Thyphimurium* isolated during 2011–2015 was more susceptible; only four of the isolates showing resistance to more than three of the antibiotic classes investigated.

The percentage of pathogenic *Salmonella* isolates resistant to three or more classes of antibiotics showed an increase in the isolates found between 2015–2021 (*n* = 34) compared to 2011–2014 (*n* = 28) (Table 2). The lowest percentage of *Salmonella* strains susceptible to at least three antimicrobials was found in the years 2020–2021 (*n* = 2). Conversely, MDR increased from 24% between 2011–2015 (*n* = 13) to 56% between 2016–2021 (*n* = 21). In addition, significant changes in the resistance patterns (*p* < 0.001) were found when comparing the pathogenic *Salmonella* isolates found in 2011–2015 and those from 2016–2021. The most marked increase in resistance was found for tetracyclines, sulfonamides and ampicillin. There was also a significant increase in resistance (Mann–Kendall test) (*p* < 0.05) in the resistance patterns for cephalosporins during the years 2016–2021. Three strains of *Salmonella* Enteritidis found in the year 2020 have shown also concurrent resistance to cephalosporin and sulphamethoxazole/trimethoprim. Two of the pathogenic *Salmonella* isolates found in 2020 in poultry carcasses have shown resistance to all antimicrobials tested. The pathogenic *Salmonella* strains isolated during the years 2011–2015 have shown a low resistance to cephalosporin and sulphamethoxazole/trimethoprim. The most common resistance phenotype of the pathogenic *Salmonella* isolates during years 2011–2015 was tetracycline (87%), nalidixic acid (52%) and ampicillin (46%). During the period of 2016–2021 we noticed a larger variety in the resistance patterns. The most common resistance phenotype was similar to that observed in the previous period but with higher percentages in prevalence (tetracycline (84%), nalidixic acid (78%) and ampicillin (78%)). Among all the *Salmonella* isolates found in our study (*n* = 91), 18 different resistance patterns were found and most of them were represented by a minimum of 3 strains. More than 24% (*n* = 22) of the isolates showed resistance to five or more antimicrobials, while only one strain belonged to the period 2011–2015.

### 2.3. Prevalence of Resistance Genes

The prevalence of resistance genes was strongly correlated to the resistance pattern found by the classical disk diffusion method. In this respect, the most common resistance genes found were those encoding for tetracycline (*tetA*, *tetB*, *tetC*) (*n* = 79) and sulphonamide (*SulI*; *SulII*) (*n* = 85). All the pathogenic *Salmonella* isolated during 2016–2021 tested positive for at least one resistance gene encoding for tetracycline resistance, with the *tetA* gene being the most prevalent (62%; *n* = 56). We also found that in the isolates from the period 2011–2015, seven of them carried the *tet* genes (*tetA*, *tetB*) but were not phenotypically resistant. Because in all isolates tested positive for one resistance gene encoding for tetracycline, *tetB* and *tetC* were also identified in a high proportion (45% and 23%, respectively). A concerning fact was that the highest number of samples that carried simultaneously the *sul1* and *tet* genes (81%; 30/37) were isolated during the period 2016–2021, which shows that there is a growing antimicrobial potential. Furthermore, 64% (24/37) of the *Salmonella* isolates from the period 2016–2021 carried at least one of the genes (*blaCMY-2*; *blaSHV1*, *blaTEM1*). In the case of genes responsible for chloramphenicol resistance, there was an obvious increase in positivity at the isolates from 2016–2021. If during the years 2011–2015 only 4 of isolates were positive to *cat1*, during the years 2016–2021, 16 samples were found positive to at least 1 of the genes *cat1*, *cat2*, *floR*. The most prevalent phenotype for chloramphenicol resistance in the positive *Salmonella* samples was *cat1*, *floR*. Only one of the samples investigated was positive for *cat2* simultaneously with *floR*. For gentamicin resistance, we investigated two incriminating genes: *aac(3)11a* and *aph(3)11a*. Only four samples were found positive for *aac(3)11a*, all of them being isolated during 2016–2021. Regarding the phenotypes of resistance, we found that the presence of multiple genes was significantly higher (*p* < 0.001) between 2016–2021 than in the years 2011–2015.

## 3. Discussion

In the past 10 years, the increasing resistance to frequently used antimicrobial agents in *Salmonella* infections has been widely reported [17]. Despite the multitude of improvements in animal food products processing in Romania and also the mandatory implementation of HACCP programs for surveillance of potential biological hazards, *Salmonella* spp. remains a leading pathogen responsible for foodborne illness. As seen in our study, the isolation of pathogenic strains of *Salmonella* was possible each year during the period studied (2011–2021) from poultry meat products. According to a study performed in Malaysia, *Salmonella* was found in a high proportion, with a rate of 35.5% and 50% in broiler carcasses at wet markets and processing plants [18]. Another study performed in Bangladesh, found the overall prevalence of *S. typhimurium* and *S. enteritidis* at a rate of 3.67% and 0.57% in chicken cecal contents [19]. The most prevalent serovar identified in the poultry meat samples investigated during the period studied was *S. enteritidis* (*n* = 56%). This finding is similar to other studies, which found a higher prevalence of *S. Enteritidis* compared to *S. thyphimurium* [20,21,22]. According to a recent global review study on the antibioresistance patterns of *Salmonella* isolated from raw chicken meat, it was found that the median prevalence was 30%, and the most prevalent serotypes were *S. enteritidis* and *S. typhimurium* [17]. Unfortunately, the presence of *Salmonella* isolates was not the only worrisome aspect, their multiple drug-resistance phenotypes were noticeable in the isolates from later years. We have found high levels of resistance to commonly used antibiotics in poultry production, such as tetracycline (84%), nalidixic acid (78%) and ampicillin (78%). These findings are in accordance with other studies performed in various countries. A previous study in Iran, revealed that the majority of the *Salmonella* isolates were resistant to nalidixic acid, tetracycline and streptomycin [23]. The resistance rates reported are in agreement with the results reported in the United States [24] and Europe [25,26,27]. The high prevalence of resistance to nalidixic acid was also reported in studies by Khaltabadi Farahani et al. (2018) (94.1/%) [28], En-Nassiri et al. (82%) (2017) [29], and Ziyate et al. (2016) (61%) [30].

Among the 91 pathogenic *Salmonella* isolates found in this study, the *SulI* gene was determined at the highest proportion (93.4%), followed by *Tet* genes (86.8%) and *blaTEM* (64%). Regarding the genes incriminated for resistance, our findings are in accordance with a recent study in Turkey, but unfortunately the frequencies of their occurrence in *Salmonella* strains show much higher levels [22]. We also found four *Salmonella* strains positive to *cat1* gene, which is responsible for chloramphenicol resistance, while many studies have shown a high level of sensitivity to this antibiotic class [22]. Overall, the prevalence of resistance genes was correlated with the phenotypical expression of the isolates, only in seven strains the presence of *Tet* genes did not show an actual resistance to this class. The gene *floR*, responsible for the resistance to florfenicol, was detected in all the strains resistant to the chloramphenicol antibiotic class. These findings are similar to those found in *Salmonella* strains isolated from various sources of raw meat [31]. The presence of *blaTEM* gene was found in 28 isolates (30.7%) and also correlated with the phenotypical expression of resistance. A previous study has shown that *blaTEM* gene sequence was present in all *Salmonella* isolates (100%), however it had low correlation (velez). For gentamicin resistance we have investigated two of the most incriminated genes: *aac(3)11a* and *aph(3)11a*. Only four samples were found positive to the presence of *aac(3)11a,* while none of the samples investigated showed the presence of *aph(3)11a.* Our findings are in contrast to previous findings, which have shown a higher prevalence of these genes and a very strong phenotypical resistance to this antimicrobial class all *Salmonella* spp. strains [32].

Our study has also revealed different resistance patterns when comparing the time intervals investigated. This statistically different pattern of resistance reported can be explained by the change in the treatment protocol. During the early years of 2000, the traditional first line *Salmonella* infection treatment was formed by chloramphenicol, ampicillin and trimethoprim-sulfamethoxazole. The high resistance rate to these classes of antibiotics in time has forced therapists to choose other classes which still had efficiency in treatment, such as cephalosporins [33]. It is concerning however that our study has revealed that new *Salmonella* strains have also begun to show resistance to these classes of antibiotics. Although the numbers are not high, the ongoing use of these antibiotics as possible treatment will eventually result in the spread of more resistant strains.

To the best of the authors’ knowledge, this is the first comprehensive study on the prevalence of the antibiotic resistance profile of pathogenic *Salmonella* isolates from poultry meat products in Romania. Our results revealed that resistance to tetracycline, nalidixic acid, streptomycin and ampicillin are high, and they must be used only when it is sure that they will be efficient.

## 4. Materials and Methods

### 4.1. Sampling

The study was conducted on 112 *Salmonella* strains isolated from poultry meat and poultry meat products during the years 2011–2021 in Romania. The strains were isolated in the Sanitary Veterinary Laboratory for Food Safety situated in the Transylvanian region of Romania, which normally receives samples for investigation. All the strains were isolated through the classical microbiological protocol recommended by the International Organization for Standardization (ISO) 6579 [34] and the positive strains were afterwards stored in freezing conditions (−80 °C). In order to perform the testing, all the strains stored were re-cultured on xylose lysine deoxycholate (XLD) agar (Oxoid, Basingstoke, UK).

### 4.2. Susceptibility Testing

All *Salmonella* isolates were tested for antimicrobial susceptibility after their isolation. The testing was performed in the Sanitary Veterinary Laboratory for Food Safety situated in Cluj County, Romania. The testing was made on eleven antimicrobials that are normally used in human and animal therapy. The method applied was the disk diffusion method and the protocol followed exactly the guidelines of the Clinical and Laboratory Standards Institute (CLSI) [35,36]. The antimicrobial classes tested were ampicillin (AMP, 10 μg), cefotaxime (CTX, 30 μg), ceftazidime (CAZ, 30 μg), chloramphenicol (CHL, 30 μg), ciprofloxacin (CIP, 5 μg), gentamicin (GEN, 10 μg), nalidixic acid (NA, 30 μg), streptomycin (S, 10 μg), sulfamethoxazole (SMX, 300 μg) trimethoprim/sulfamethoxazole (SXT, 1.25/23.75 μg), and tetracycline (TET, 30 μg) (Oxoid, Basingstoke, UK). The interpretation of the results was made according to the CLSI breakpoints.

### 4.3. DNA Extraction Protocol

The bacterial DNA was extracted following a simple protocol previously described by Mihaiu et al. (2014) [37]. Briefly, we selected from the XLD medium, two or three specific colonies that had the black center specific to pathogenic strains. The colonies were taken with a microbiological loop and suspended into Eppendorff tubes (1.5 mL) (Ratiolab, Dreieich, Germany) that contained 150 μL of CHELEX (10%) reactive (BioRad, Berkeley, CA, USA). The following extraction temperatures were used: 57 °C-30′; 94 °C-5′. The last step included a high-speed centrifugation (14.000 rotations per minute) for one minute.

### 4.4. PCR Protocol for Confirmation of Pathogenic Strains

All the samples were tested for confirmation of the pathogenic strains *Salmonella typhimurium* and *Salmonella enteritidis.* We carried out a PCR multiplex, using a specific protocol previously described by Mihaiu et al. (2014) [37]. For the positive control, *Salmonella typhimurium* ATCC 14,028 and *Salmonella enteritidis* ATCC 13,076 strains were used.

### 4.5. PCR Protocol for Resistance Gene Testing

In order to detect the antimicrobial resistance patterns of the pathogenic strains, the study also focused on identification of the major genes that conferred resistance to the classes of antibiotics studied through disk diffusion method. The genes and specific primers are listed in Table 3. Briefly, the PCR was performed in a 25 µL reaction mix that comprised: 1×PCR green Buffer, 2.5 mM MgCl_2_, 5 pmol of each primer, dNTPs each at 200 μM, 2.5 U of TaqDNA polymerase (Promega), and 100 ng of genomic DNA. The PCR was performed under the following conditions: 94 °C for 3 min followed by 35 cycles of 94 °C for 30 s, 55 °C for 30 s, and 72 °C for 1 min, and a final extension step of 73 °C for 5 min. A total of 10 μL from each PCR reaction containing the amplified product was loaded onto agarose gels (2%). The gels were stained with EvaGreen (JenaBioscience, Jena, Germany) and electrophoresed (90 W) for 40 min. Visualization was performed under UV light with a Gel Doc XR+Imager (Bio-Rad, Hercules, CA, USA).

### 4.6. Statistical Analysis

In order to identify the overall trends in antimicrobial susceptibility of the strains isolated over a long period of time (10 years), the Mann–Kendall test was performed. The magnitude of the possible changes during the 10 years was estimated using a slope parameter (Q) and the Sen non-parametric method, according to a previously described protocol [38]. The ten-year period was analyzed according to two time intervals (2011–2015; 2016–2021) taking into account the number of samples recovered and the detection of possible changes in resistance. In this respect, between 2011–2015, 54 samples were investigated and 37 samples in the second time interval. Significance was assessed at *p* < 0.05.

**Table 3:** Primer sequences and their annealing temperatures.

Gene	Nucleotide Sequence 5′–3′	Product Size (pb)	Annealing t (°)	Antibiotic	Reference
*tetA*	F:TTGGCATTCTGCATTCACTC R:GTATAGCTTGCCGGAAGTCG	494	55	TET	[39]
*tetB*	F:GTATAGCTTGCCGGAAGTCG R:CAGTGCTGTTGTGTCATTAA	571	55	TET	[39]
*tetC*	F:GCTTGGAATACTGAGTGTAA R:CTTGAGAGCCTTCAACCCAG	418	55	TET	[39]
*Sul1*	F:CAAAGCCCCTTGCTTGTTAC R:TTTCCTGACCCTGCGCTCTAT	793	55	SMX, SXT	[40]
*Sul2*	F:GTGCGGACGTAGTCAGCGCCA R:CCTGTTTCGTCCGACACAGA	667	55	SMX, SXT	[40]
*cat1*	F:AACCAGACCGTTCAGCTGGAT R:CCTGCCACTCATCGCAGTAC	549	55	CHL	[41]
*cat2*	F:AACGGCATGAACCTGAA R:ATCCCAATGGCATCGTAAAG	547	55	CHL	[40]
*floR*	F:ATGACCACCACACGCCCCG R:AGACGACTGGCGACTTCTTCG	198	55	CHL	[40]
*aac(3)11a,*	F:CGGCCTGCTGAATCAGTTTC R:AAAGCCCACGACACCTTCTC	439	55	GEN	[39]
*aph(3)11a*	F:TCTGAAACATGGCAAAGGTAG R:AGCCGTTTCTGTAATGAAGGA	582	55	GEN	[39]
*blaCMY-2*	F:TGG CCG TTG CCG TTA TCT AC R:CCC GTT TTA TGC ACC CAT GA	870	55	β-Lactams	[42]
*blaSHV-1*	F:GGC CGC GTA GGC ATG ATA GA R:CCC GGC GAT TTG CTG ATT TC	714	55	β-Lactams	[42]
*blaTEM-1*	F:CAG CGG TAA GAT CCT TGA GA R:ACT CCC CGT CGT GTA GAT AA	643	55	β-Lactams	[42]

## 5. Conclusions

In conclusion, the present study revealed the concerning increase of antimicrobial resistant *Salmonella* in poultry meat products during a ten-year period of time, with a particular emphasis on the occurrence of antimicrobial resistance genes. During the years 2016–2021, a high incidence of *Salmonella* exhibiting MDR phenotypes was noticed. The high rate of resistance genes in these isolates, particularly those encoding for sulfonamides and β-lactams resistance suggested that the *Salmonella* surveillance system needs to be more strictly applied in poultry production. The study shows growing evidence that *Salmonella* bacteria will become increasingly difficult to treat and that more strict regulations are urgently needed to limit antimicrobial use. We suggest that the studies should be extended to *Salmonella* isolates along the food chain in order to furtherly explore and quantify the epidemiological situation and to spread the extent in the environment.

## Figures and Tables

**Table 1:** Antimicrobial susceptibility of *Salmonella* isolates in poultry meat and poultry meat products in the period 2011–2015 54/38;16.

Sample No./Year	Serotype	Product	Antimicrobial Resistance Profile	Antimicrobial Resistance Genes
1,2,3,4,5,6,7/2011	*S. enteritidis*	Poultry meat (carcass)	S, TET	*sulI, tetA*
8,9,10,11/2011	*S. enteritidis*	Poultry meat (chicken breast)	NA, TET	*tetA, tetB*
12,13/2012	*S. enteritidis*	Poultry meat (carcass)	S, TET	*sulI, dfr, tetA*
14/2012	*S. thyphimurium*	Poultry meat (carcass)	AMP, TET,	*sulI, sulII, tetA,*
15,16,17,18/2013	*S. enteritidis*	Poultry meat (carcass)	SMX, TET	*sulI, tetB, tetC*
19,20,21/2013	*S. thyphimurium*	Poultry meat (carcass)	CHL, AMP, TET, NA	*sulI, sulII, tetA, tetB, cat1*
22,23,24,25/2013	*S. enteritidis*	Poultry meat (carcass)	CAZ, NA, TET, S	*sulI, sulII, tetA, tetC*
26,27,28/2013	*S. enteritidis*	Poultry organs (liver)	SMX, AMP, TET, NA	*sulI, sulII, tetA, tetB,*
29,30/2014	*S. enteritidis*	Poultry meat (carcass)	TET, NA	*sulI, tetB, tetA*
31,32,33/2014	*S. thyphimurium*	Poultry meat (carcass)	S, AMP	*sulI, sulII, tetA, tetB*
34,35/2014	*S. enteritidis*	Poultry organs (liver)	AMP, NA, S, CAZ	*dhfr1, sulI, tetA*
36,37,38,39/2014	*S. enteritidis*	Poultry organs (liver)	NA, TET	*blaTEM-1, aadA1, sulI, sulII, tetA, tetB*
40,41,42/2014	*S. thyphimurium*	Poultry meat (breast)	AMP, TET	*aadA1, sulI, sulII, tetB,*
43,44/2014	*S. thyphimurium*	Poultry meat (chicken wings)	NA, TET	*aadA1, sulI, sulII*
45,46/2014	*S. enteritidis*	Poultry organs (hearts and gizzards)	NA, AMP	*blaTEM-1, aadA1, dhfr1, sulI, tetA*
47,48/2015	*S. enteritidis*	Poultry meat (carcass)	AMP, TET	*aadA1, sulI, sulII, tetB*
49/2015	*S. thyphimurium*	Poultry meat (carcass)	CHL, AMP, TET, SXT, NA	*aadA1, sulI, sulII, tetA, tetB, tetC, cat1*
50,51,52,53/2015	*S. enteritidis*	Poultry meat (carcass)	AMP, TET	*aadA1, sulI, sulII, tetB,*
54/2015	*S. thyphimurium*	Poultry organs (liver)	NA, TET	*sulI, sulII, tetA*

**Table 2:** Antimicrobial susceptibility of *Salmonella* isolates in poultry meat and poultry meat products in the period 2016–2021.

Sample No./Year	Serotype	Product	Antimicrobial Resistance Profile	Antimicrobial Resistance Genes
55/2016	*S. enteritidis*	Poultry organs (hearts and gizzards)	NA, TET	*sulI, sulII, tetA, tetB, tetC*
56/2017	*S. enteritidis*	Poultry meat (carcass)	AMP, TET	*aadA1, sulI, tetB, tetA, tetC*
57/2017	*S. thyphimurium*	Poultry meat (carcass)	NA, AMP, TET, CAZ	*aadA1, dhfr1, sulI, sulII, tetA, cat2*
58,59,60/2018	*S. enteritidis*	Poultry meat (carcass)	SMX, NA, GEN, AMP, TET, SXT	*blaCMY-2, blaTEM-1, aadA1, aac(3)11a, sulI, sulII, tetA, tetC*
61,62/2018	*S. enteritidis*	Poultry meat (chicken breast)	S, NA, AMP, TET, CHL	*aadA1, sulI, sulII, tetB, blaTEM-1, cat1, floR*
63,64/2018	*S. thyphimurium*	Poultry meat (carcass)	NA, TET	*sulI, sulII, tetB*
65/2018	*S. thyphimurium*	Poultry meat (carcass)	S, AMP, TET, SXT, CAZ, GEN	*aadA1, sulI, sulII, tetB, blaTEM-1, cat1. aac(3)11a*
66/2018	*S. thyphimurium*	Poultry organs (liver)	SUL, TET	*sulI, tetA, tetB, tetC*
67,68/2019	*S. enteritidis*	Poultry meat (carcass)	SMX, S, AMP, TET, SXT, CHL	*aadA1, sulI, sulII, tetB, blaTEM-1, cat1*
69,70/2019	*S. enteritidis*	Poultry meat (carcass)	NA, TET	*blaTEM-1, sulI, tetA*
71/2019	*S. enteritidis*	Poultry organs (liver)	AMP, TET	*aadA1, sulI, sulII, tetB, blaTEM-1*
72,73/2020	*S. enteritidis*	Poultry meat (carcass)	S, AMP, TET, CIP, SXT, CHL	*aadA1, sulI, tetA, tetB, tetC, blaTEM-1, dhfr1, cat1*
74,75/2020	*S. thyphimurium*	Poultry meat (carcass)	NA, TET,	*sulI, sulII, tetA, tetB, tetC*
76,77,78/2020	*S. enteritidis*	Poultry organs (liver)	AMP, NA	*dhfr1, sulI*
79,80/2020	*S. enteritidis*	Poultry organs (liver)	SMX, NA, S, AMP, TET, SXT	*blaCMY-2, blaTEM-1, aadA1, dhfr1, sulI, sulII, tetA,*
81,82,83/2020	*S. thyphimurium*	Poultry meat (breast)	SMX, S, AMP, TET, SXT, NA, CHL	*aadA1, sulI, sulII, tetB, blaTEM-1, cat1*
84,85/2021	*S. thyphimurium*	Poultry meat (chicken wings)	AMP, NA	*aadA1, dhfr1, sulI, sulII*
86,87,88/2021	*S. enteritidis*	Poultry meat (carcass)	S, AMP, NA, TET, SXT, CHL	*aadA1, sulI, tetA, tetB, tetC, blaTEM-1, dhfr1, cat1, floR*
89,90/2021	*S. enteritidis*	Poultry organs (liver)	SMX, S, AMP, NA, TET, SXT, CHL	*aadA1, sulI, sulII, tetB, blaTEM-1, cat1, cat2, floR*
91/2021	*S. enteritidis*	Poultry meat (carcass)	S, AMP, TET, CIP, SXT, NA, CHL	*aadA1, sulI, tetA, tetB, tetC, blaTEM-1, dhfr1, cat1, floR*
